# Disproportionate Distribution of HBV Genotypes A and D and the Recombinant Genotype D/E in the High and Low HBV Endemic Regions of Uganda: A Wake-Up Call for Regional Specific HBV Management

**DOI:** 10.1155/2022/3688547

**Published:** 2022-01-11

**Authors:** Hussein Mukasa Kafeero, Dorothy Ndagire, Ponsiano Ocama, Charles Drago Kato, Eddie Wampande, Henry Kajumbula, David Kateete, Abdul Walusansa, Ali Kudamba, Kigozi Edgar, Fred Ashaba Katabazi, Maria Magdalene Namaganda, Jamilu E. Ssenku, Hakim Sendagire

**Affiliations:** ^1^Department of Microbiology, Makerere University, College of Health Sciences, P. O. Box 7062, Kampala, Uganda; ^2^Department of Medical Microbiology, Habib Medical School, Faculty of Health Sciences, Islamic University in Uganda, P.O. Box 7689, Kampala, Uganda; ^3^Department of Plant Sciences, Microbiology and Biotechnology, College of Natural Sciences, Makerere University, P.O. Box 7062, Kampala, Uganda; ^4^Department of Medicine, College of Health Sciences, Makerere University, P.O. Box 7062, Kampala, Uganda; ^5^Department of Biomolecular Resources & Biolab Sciences (BBS) College of Veterinary Medicine, Animal Resources and Biosecurity (COVAB), Makerere University, P.O. Box 7062, Kampala, Uganda; ^6^Department of Molecular Biology and Immunology, College of Health Sciences, Makerere University, P.O. Box 7062, Kampala, Uganda; ^7^Department of Physiology, Habib Medical School, Faculty of Health Sciences, Islamic University in Uganda, P.O. Box 7689, Kampala, Uganda; ^8^Department of Biological Sciences, Faculty of Science, Islamic University in Uganda, P.O. Box 2555, Mbale, Uganda

## Abstract

**Background:**

Hepatitis B virus (HBV) is the leading cause of liver-related diseases. In Uganda, there is a regional disparity in the HBV burden. Our study was aimed at establishing the circulating genotypes in a low and a high endemic region to give plausible explanations for the differences in regional burden and guide the future management of the disease.

**Methods:**

A total of 200 HBsAg-seropositive subjects were recruited into the study by convenience sampling. The HBsAg Rapid Test Strip (Healgen Scientific Limited Liability Company, Houston, TX77047- USA) was used to screen for HBsAg while the Roche machine (Roche, Basel Switzerland/Abbot Technologies (USA)) was used to determine the viral load. The Chemistry Analyzer B120 (Mindray, China) was used for chemistry analysis. For HBV genotyping, total DNA was extracted from whole blood using the QIAamp® DNA extraction kit. Nested PCR amplification was performed using Platinum Taq DNA Polymerase (Invitrogen Corporation, USA) to amplify the 400 bp HBV polymerase gene. Purification of nested PCR products was performed using Purelink PCR product purification kit (Life Technologies, USA). Automated DNA sequencing was performed using BigDye Terminator v3.1 Cycle Sequencing Kit on 3130 Genetic Analyzer (Applied Biosystems, USA). The NCBI HBV genotyping tool (https://www.ncbi.nlm.nih.gov/projects/genotyping/formpage.cgi) was used for determination of genotype for each HBV sequence. Pearson's chi-square, multinomial logistic regression, and Mann–Whitney *U* tests were used for the analysis. All the analyses were done using SPSS version 26.0 and MedCalc software version 19.1.3 at 95% CI. A *p* < 0.05 was considered statistically significant.

**Results:**

Majority of our study subjects were female (64.5%), youth (51.0%), and married (62.0%). Overall, genotype A was the most prevalent (46%). Genotype D and the recombinant genotype D/E were proportionately more distributed in the high endemic (38.2%) and low endemic (36.5%) regions, respectively. Genotype D was significantly more prevalent in the high endemic region and among the elderly (*p* < 0.05). Genotype D was significantly associated with elevated viral load and direct bilirubin (*p* < 0.05). The recombinant genotype D/E was significantly associated with elevated viral load (*p* < 0.05). Similarly, genotype A was significantly associated with elevated AST and GGT, lowered viral load, and normal direct bilirubin levels (*p* < 0.05).

**Conclusion:**

There is disproportionate distribution of genotypes A and D and the recombinant genotype D/E in the low and high endemic regions of Uganda. This probably could explain the differences in endemicity of HBV in our country signifying the need for regional specific HBV management and control strategies.

## 1. Background

Hepatitis B virus (HBV) infects the liver causing acute hepatitis which can progress to chronicity in those who fail to resolve the infection within 6 months [1]. The global burden of the disease is estimated at 257 million chronic infections with Western Pacific (6.2%) and Africa (6.1%) having the highest burden [2]. In east Africa, an HBV prevalence rate of 6.025% has been reported [3]. In Uganda, the prevalence in the general population is estimated at 4.3% with differences in regional burden. The northern region has the highest prevalence followed by midnorth and West Nile at prevalence rates of 4.6%, 4.4%, and 3.8%, respectively. Other regions have low prevalence ranging from 0.8% in south-west, 1.9% in midwest, 2.0% in central, and 2.3% in mideast [4]. The reasons for the regional differences in the endemicity are not fully understood but studies elsewhere have implicated the infecting genotypes [1, 5, 6].

Globally there are ten genotypes A-J based on the divergence of ≥8% in the complete genome and several subgenotypes [7]. Genotype A has three subgenotypes (A1-3), Genotype B has six subgenotypes B1-6, genotype C has five subgenotypes C1-5, genotype D has five subgenotypes D1-5, and genotype E has no reported subgenotype up to date [8]. In Uganda, six genotypes have been reported; A, B, C, D, E, and G, by Zirabamuzaale and Ocama [9]. The HBV genotypes have been found to influence disease burden and response to antiviral therapy [1, 7]. For example, poor response to INF-*α* treatment in patients infected with genotypes C and D compared to those infected with genotypes A and B has been reported [10, 11]. Similarly, an increased likelihood of resistance to nucleotide/nucleoside analog antivirals but better response to interferons has also been reported among patients infected with genotype A [8]. Consequently, in areas where HBV patients are predominantly infected with genotype A, there is an increased risk of developing chronic disease that is resistant to nucleotide analog [12]. Besides, the variations in response to antiviral therapy with the infecting genotype and natural disease prognosis has also been associated with particular genotypes. For example, genotype C has been associated with poorer prognosis than B with a higher risk of hepatocellular carcinoma (HCC) [10, 11, 13]. Nonetheless, other studies have implicated genotype B in poorer prognosis than C [14, 15]. Additionally, genotype A has been frequently associated with progression to chronic infection [8, 16] and HCC in African population [16] compared to other genotypes. Thus, in African populations with high prevalence of genotype A, there is an increased risk of progression to chronic disease as well as hepatocellular carcinogenicity [12].

Particular genotypes have also been associated with elevated viral load, increasing the risk of HCC as well as post-HCC treatment recurrence. For example, infection with genotype C is associated with higher HBV DNA levels (viral load) than B and genotype D higher than A [17–20]. Similarly, higher viral loads in patients infected with genotype mixtures or recombinant genotypes compared to those infected with a single genotype have been reported [21]. Besides, viral load influences staging of the disease and response to antiviral therapy and increases the risk transmission of the virus from the mothers to their children [9, 22]. Nonetheless, the study by Tran et al. [23] did not establish an obvious relationship between viral load and genotype. There is also evidence implicating particular genotypes to the route of transmission. Genotype A is associated with sexual transmission, D with blood transfusion, B and C with mother-to-child transmission, and G with homosexual transmission in a coinfection with genotype H or A [11, 15, 24–26].

Unfortunately, evaluation of genotypes is not part of HBV routine care in most of the developing countries [27, 28] where the burden of the disease is overwhelmingly high. In Uganda, there has been no routine care among the HBV-infected persons. It is only recently that care programs such as testing, vaccination programs among the high-risk groups like health workers, and testing for viral load before treatment candidacy is established among others have been initiated. Even with the recent awareness campaigns of HBV as a major public health problem in the country, genotyping is not part of the routine care. Thus, little is known about the circulating genotypes in our country. Understanding the circulating genotypes in a Ugandan population will give constructive clues on the plausible routes of transmission, suitable antiviral therapeutic regimens, viral load status among the chronically infected persons, the risk of progressing to liver carcinoma, and consequently the variation in regional disease burden. The main aim of this study was to determine and compare the hepatitis B virus genotypes from high and low endemic regions in order to draw conclusions regarding underlying reasons for the regional differences in the burden of the disease.

## 2. Materials and Methods

### 2.1. Ethical Approval

The study obtained ethical approval from the Research and Ethics Committee (REC) of the School of Biomedical Sciences (SBS), College of Health Sciences, Makerere University, reference number SBS-REC-708, and the Uganda National Council for Science and Technology (UNCST), reference number HS575ES.

### 2.2. Study Design

This was a cross-sectional laboratory-based study.

### 2.3. Study Site

**Figure 1:**
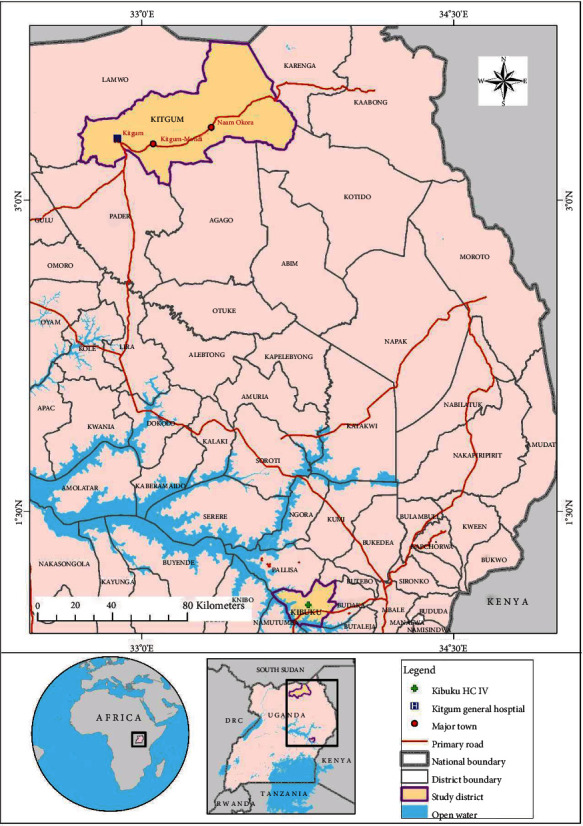
Study sites from both low and high endemic regions and the neighboring catchment areas.

In the Uganda Population-Based HIV-Impact Assessment (UPHIA) study, the high endemic regions were in northern Uganda, West Nile, mid-north, and north-east while the low endemic regions were in south-west, midwest, central, mideast, and east-central. So, we recruited from both high and low endemic regions (Figure 1). According to our study, we conventionally defined the low and high endemic regions in reference to the national HBV prevalence of 4.3%. By default, any region with a prevalence less than the national prevalence of 4.3% merited inclusion in the study as a low endemic region. In contrast, any region with a prevalence greater than 4.3% warranted selection for inclusion in the study. Basing on the aforementioned criteria, participants from Kitgum and the neighboring districts (Lamwo, Karenga, Pader, Agago, and Kotido districts) that form part of the midnorth were recruited in the study to represent the high endemic region. Kitgum and the neighboring districts form the midnorth corridor with the highest burden of the disease in our country and were purposively chosen. Similarly, from the low endemic region, we recruited from Kibuku and the surrounding districts (Butebo, Budaka, Butaleja, Namutumba, and Pallisa) that form part the mideastern region with a prevalence of 2.3%. This region has a relatively high prevalence within the southern corridor that has continued to post low prevalence and was purposively chosen to increase the chances of HBsAg-seropositive samples [4].

### 2.4. Populations and Study Units

All the HBsAg-seropositive individuals from regions with high and low HBV endemicity constituted our target population. Similarly, the HBsAg-seropositive individuals from the selected districts and the neighboring catchment areas constituted our study population from whom the study units were derived. The study units were participants, 18 years and above, who participated in the national screening program in Kibuku district where immunization program was ongoing during the time of sample collection. In Kitgum district where the national screening program had been completed, those who participated were contacted from the hospital or health centre records. New cases that never participated in the screening program coming to the hospitals/health facilities for routine screening and or viral load evaluation were also recruited if found HBsAg seropositive. These gave written informed consent to participate in the study.

### 2.5. Sample Size Determination and Sampling Procedure

For analysis of the circulating genotypes in the low and high hepatitis B endemic areas, the formula for calculating sample size in prevalence studies was used as described by Cochran [29]. Using the national prevalence of 4.3% in the general population at 95% level of significance and a precision of 5%, a sample size of 70 chronic cases from the low endemic region was used after allowing for a 10% increase due to patients with inadequate blood. Another sample of 65 chronic cases from the high endemic region was used giving a sample size of 135 for the investigation of the circulating hepatitis B genotypes in the low and high endemic regions. However, to cater for the likelihood of undetectable HBV DNA during the polymerase chain reaction assay, despite the HBsAg seropositivity, we extracted HBV DNA from 101 samples from the low and 99 samples from the high endemic regions giving a total of 200 samples. Convenient sampling was used by screening volunteer hospital outpatients for HBsAg and recruiting those who tested positive at both study sites until when the required sample size was attained. The sociodemographic characteristics of the HBsAg-seropositive persons as well as those whose samples gave clear bands after visualization of ethidium bromide-stained gels under ultraviolet light were documented.

### 2.6. Exclusion and Inclusion Criteria

A two-stage eligibility criterion was done. First, all participants who tested positive for the HBsAg at screening during their first visit to the health facility and those already positive and coming for review as well as those coming for viral load evaluation at Kibuku Health Center IV and Kitgum General Hospital were included in our study. In contrast, those screened for HBsAg and were found to be seronegative were excluded from the study. Second, only participants whose sample had detectable HBV DNA after screening were included in the analysis of the infecting genotypes. On the other hand, persons with undetected HBV DNA were excluded from the analysis of HBV genotypes.

### 2.7. Sample Collection

4 mL of venous blood was collected by a trained laboratory technician in purple top vacutainers and kept at -20°C pending sample transportation to the molecular biology laboratory of the school of Biomedical Sciences, College of Health Sciences, Makerere University, where they were frozen at -80°C until HBV DNA was extracted. In addition, 4 mL of venous blood was collected in red top vacutainers and left at room temperature for serum to separate out which was decanted off in viols as soon as clear serum was separated from the cells to avoid hemolysis which would otherwise give false elevations in *γ*GT and ALP. This was kept at -20°C for chemistry parameters analysis and viral load quantification.

### 2.8. Study Setting

This was a laboratory-based study conducted from three laboratories: Molecular Biology Laboratory, Department of Immunology and Molecular Biology, Makerere University College of Health Sciences for molecular assays, Ministry of Health Republic of Uganda; Central Public Health Laboratory Services/Uganda National Health Reference laboratories (CPHL/UNHL), Butabika for viral load assay; and the Kibuli Muslim Hospital Laboratory; clinical chemistry assays were conducted at the teaching hospital of the Habib Medical School (HMS), Faculty of Health Sciences (FHS), Islamic University in Uganda (IUIU).

### 2.9. Statistical Analysis

Descriptive statistics was used to report the frequency and distribution of particular genotypes in low and high endemic areas. The results were presented in frequency tables and bar graphs and then reported as proportions of the total samples that were successfully sequenced. A bilevel analysis was done to comprehensively establish whether a statistical association between the dependent variables (DV) of viral load, sociodemographic characteristics, predictors of HBV infection, and the chemistry parameters with the independent variable (IV) of genotypes existed or not. First, the bivariable analysis was performed by using the Pearson chi-square to select candidate variables for the inclusion in the final model of analysis. Second, the multinomial logistic regression analysis was used for the final analysis of any association between the DV and IV. The normality in the data was tested for by using D'Agostino-Pearson test. The association of the mean levels of the clinical chemistry parameters and viral load with HBV genotypes was analyzed by using the parametric Whitney *U* test due to lack of normality in the data. All analyses were done at 95% level of significance using SPSS version 26.0 and MedCalc statistical software version 19.1.3. A *p* < 0.05 was considered statistically significant.

### 2.10. Serology, Viral Load Testing, and Clinical Chemistry Parameter Assays

Screening for HBsAg seropositivity was done using the HBsAg Rapid Test Strip (Healgen Scientific Limited Liability Company, Houston, TX77047, USA) that uses lateral flow chromatographic immunoassay for the qualitative detection of Hepatitis B Surface Antigen (HBsAg) in the human serum or plasma based on the double-antibody sandwich technique. The Roche Molecular Systems, Pleasanton, CA, USA HBV assay (Abbott Molecular, Des Plaines, IL, USA), with a limit of detection of 10 international units per milliliter (IU/mL) or less was used to detect and quantify the HBV DNA levels. This platform detects the HBV in the patient's serum irrespective of the genotype or sequence polymorphisms. The Roche HBV Test uses a polymerase chain reaction- (PCR-) based assay that is run on the automated cobas® 4800 System. The protocol previously described by Chevaliez et al. [30] was used. Briefly, the HBV DNA was extracted from 1000 *μ*L of plasma by use of the Cobas AmpliPrep automated extractor following the manufacturer's directions. The Cobas TaqMan 96 analyzer was used for automated real-time PCR amplification and detection of the PCR products according to the manufacturer's instructions. The data were analyzed with Amplilink software. The HBV DNA levels were expressed in international units per milliliter. The cutoff 20,000 IU/L was used in the current study because the currently used algorithm for treatment decision by the Ugandan Ministry of Health is a viral load of ≥20,000 IU/mL [31]. The liver enzymes, including alanine aminotransferase (ALT), *γ*-glutamyl transferase (*γ*-GT), alkaline phosphatase (ALP), and aspartate aminotransferase (AST) as well as albumin and bilirubin, were evaluated by standard methods using the Chemistry Analyzer B120 (Mindray, China). Normal values were considered as <30 U/L for AST, ≤40 U/L for ALT, ≤48 U/L for GGT, 30-120 for ALP, 66-83 g/L for ALB, ≤17 *μ*mol/L for total bilirubin, and ≤7 *μ*mol/L for direct bilirubin. Screening for HBsAg was done on the spot during sample collection by the laboratory technician whereas the chemistry parameters were analyzed at Kibuli Muslim Hospital Laboratory, the teaching hospital of the Habib Medical School (HMS), Faculty of Health Sciences (FHS), Islamic University in Uganda (IUIU), whereas viral load testing was done at the Central Public Health Reference Laboratories/Uganda National Health Reference Laboratories (CPHL/UNHL).

### 2.11. DNA Isolation and PCR Amplification of the HBV Polymerase Gene

Total DNA was extracted from 200 *μ*L of the whole blood using the QIAamp® DNA extraction kit following the manufacturer's instructions. Briefly, 20 *μ*L QIAGEN Protease (or proteinase K) was pipetted into a 1.5 mL microcentrifuge tube and 200 *μ*L of the whole blood sample added to the microcentrifuge tube followed by 200 *μ*L buffer AL to the sample and then mixed by pulse-vertexing for 15 s. The mixture was incubated at 56°C for 10 min. 200 *μ*L ethanol (96–100%) was added to the sample and mixed again by pulse-vertexing for 15 s. The mixture was then carefully applied to the QIAamp Mini spin column (in a 2 mL collection tube), and the cap gently closed and centrifuged at 6000 × g (8000 rpm) for 1 min, and the supernatant discarded. 500 *μ*L buffer AW1 was added to the tube and then centrifuged at 6000 × g (8000 rpm) for 1 min; then, the supernatant was discarded; this was followed by 500 *μ*L Buffer AW2, and the mixture was centrifuged at full speed (20,000 × g; 14,000 rpm) for 3 min, and the supernatant discarded and then centrifuged at full speed for 1 min to eliminate the chance of possible Buffer AW2 carryover. The supernatant was discarded, and 200 *μ*L Buffer AE added. The contents were incubated at room temperature for 1 min and then centrifuged at 6000 × g (8000 rpm) for 1 min.

The PCR amplification was performed using Platinum Taq DNA Polymerase (Invitrogen Corporation, USA). The fragment of the polymerase gene was amplified by nested PCR with two rounds of amplification. 5 *μ*L of DNA isolated from each patient was added to a 20 *μ*L PCR mixture. The PCR mix contained 2.5 *μ*L of 10x PCR buffer (100 mM Tris—pH 9.0, 500 mM KCl, 15 mM MgCl_2_, and 0.1% gelatin), 200 *μ*M dNTPs, 1 unit of Platinum Taq DNA polymerase, 20 pM each of YMDDF1 and YMDDR1 primers for first round PCR, and sterile nuclease-free water to make a final volume of 25 *μ*L.

For the first round PCR, the primers YMDDF1 (5′-CAAGGTATGTTGCCCGTTTG-3′) and YMDDR1 (5′-CCCAACTCCTCCCAGTCCTTAA-3′) previously described by Chen et al. [32] and expected to give an amplicon size of 1290 were used. The nested PCR primer pair YMDDF2 (5′–CTGTATTCCCATCCCATCATC-3′) and YMDDR2 (5′ GACCCACAATTCGTTGACATAC-3′) previously described by Chavan et al. [33] were used and gave an amplicon of 400 bp for the second round. Amplification was achieved in a Bio-Rad T100 Thermal cycler (Bio-Rad Laboratories Inc., Singapore). The first round of amplification was performed with an initial 5 min denaturing step at 95°C, followed by 30 cycles of denaturing for 45 s at 94°C, annealing for 30 s at 60°C, and elongation for 1 min at 72°C, with a final extension period of 10 min at 72°C. The second round of amplification was performed using 20 pM each of YMDDF2 and YMDDR2 primers, with an initial 5 min denaturing step at 94°C, followed by 30 cycles of denaturing for 45 s at 94°C, annealing for 30 s at 55°C, and elongation for 30 s at 72°C, with a final extension period of 10 min at 72°C. Five microliters each of the PCR product was analyzed on a 1% agarose gel stained with ethidium bromide (0.5 *μ*g/mL). Gels were run at 120 V for 1 hour and visualized using a UVP Gel documentation (Benchtop Transilluminator System, BioDoc-it, CA, USA). The reaction products of the nested PCR were visualized on a 2% (*w*/*v*) agarose gel stained with ethidium bromide.

### 2.12. DNA Purification and Sequencing of HBV Polymerase Gene

For DNA purification, we added 750 *μ*L of chloroform: isoamyl alcohol mixture to the samples and then centrifuged for 10 minutes at 16,000 × g. The partitioned DNA in the aqueous layer was transferred with care to a 1.5 mL centrifuge tube (Eppendorf Inc.) prelabeled with the sample identification number, and then, 600 *μ*L of ice-cold absolute isopropanol added (Fisher Scientific, USA) to precipitate the DNA. The mixture was kept at -20°C for 2 hours prior to centrifugation for 10 min at 16,000 × g to deposit the DNA. The deposited DNA was then washed with 1 mL of 70% ice-cold ethanol and left to dry at room temperature for 1 hour. Finally, the DNA was eluted in 50 *μ*L of 0.25X TE buffer. The quality of the extracted and purified DNA for subsequent use as a template in PCR reactions was established by electrophoresis on a 1.5% (*w*/*v*) agarose gel, and its amount determined by using a NanoDrop spectrophotometer (ThermoFisher Scientific). Automated DNA sequencing was performed using BigDye Terminator v3.1 Cycle Sequencing Kit on 3130 Genetic Analyzer (Applied Biosystems, USA). Nucleotide sequences were submitted to the National Center for Biotechnology Information (NCBI) nucleotide sequence database GenBank.

### 2.13. Determination of the HBV Genotypes

The NCBI HBV genotyping tool (https://www.ncbi.nlm.nih.gov/projects/genotyping) was used for determination of genotype for each HBV sequence. FASTA format files were picked from BioEdit software and pasted into nucleotide blast to verify the relatedness and accuracy of the sequence of the unknown target after trimming off the artefacts at the start of the chromatograms and near the end as a result of poor-quality chromatogram due to regent depletion generating approximately 380 bases. The nucleotide blast (NCBI) perfectly matches the unknown targeted sequence obtained from the sanger sequence against the known deposited sequences at a global level. These generated alignments against score values depending on the degree of relatedness of the unknown sequence against the reference sequences. In addition to relatedness, this particular alignment was aimed at generating genotypes for each unknown sequence; henceforth, the perfectly matching targets/reference genotypes were considered to be 100% related to the unknown deposited with subsequent *e* value of ≤0.

## 3. Results

Overall, serum/whole blood was obtained from 200 HBsAg-seropositive participants. Of these, 99 (49.5%) and 101 (50.5%) were from the high and the low endemic regions, respectively. Majority of the participants were female (64.5%), aged between 18 and 30 years (51.0%), married (62.0%), and had basic primary education (40.5%) (Supplementary material S1, Table A). 135 (67.5%) samples were PCR positive of which 70 (51.9%) and 65 (48.1%) were from the high and low endemic regions, respectively. 87 (63.0%) were female, 62 (45.9%) were youth aged 18-30 years, 86 (63.7%) were married, and 50 (37.0%) had the basic primary education (Table 1).

### 3.1. PCR Amplification of HBV Polymerase Gene

Hepatitis B virus DNA isolated from patients generated 400 bp PCR amplification product for polymerase gene using nested PCR approach for samples from both the low and high endemic regions (Supplementary material S2, Figures A and B).

### 3.2. Sequencing of the PCR Product to Determine the HBV Genotypes

**Figure 2:**
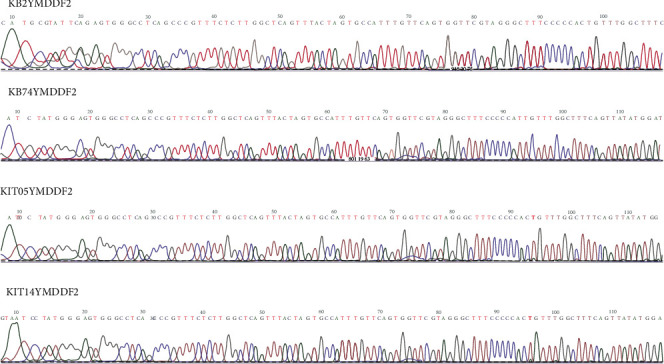
Graphs showing FASTA format sequences with the corresponding chromatograms from BioEdit for representative sequences KB2YMMDF2, KB74YMMDF2 (for Kibuku), KIT05YMMDF2, and KIT14YMMDF2 (for Kitgum).

Overall, 131 (97.0%) nested PCR product out of the 135 samples were successfully sequenced: 63 (48.1%) from the low endemic region and 68 (51.9%) from the high endemic region (Table 2, Figure 2). Only 4 (3.0%) PCR amplicons did not generate sequences: 2 (3.1%) from the high endemic region and 2 (2.9%) from the low endemic region (Table 2).

The nucleotide sequence data was submitted to GenBank; these sequences are available at NCBI nucleotide sequence database with their respective accession numbers.

### 3.3. Circulating HBV Genotypes and Subgenotypes in the Low and High Endemic Regions of Uganda

Overall, two genotypes (A and D), two subgenotype (A1 and D4), and the recombinant genotype D/E were detected as predicted by NCBI genotyping tool for HBV from the samples. Genotype prediction from NCBI genotyping tool, Geno2Pheno, and HBVSeq produced 100% similar results.

**Figure 3:**
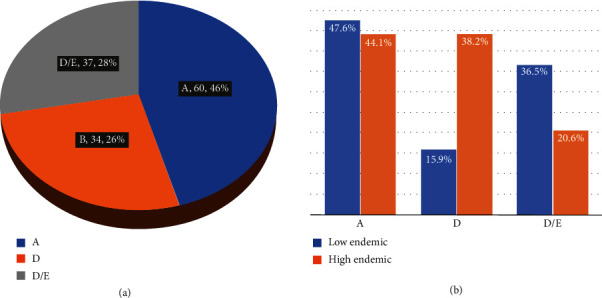
Genotype prevalence: (a) overall prevalence of the circulating genotypes; (b) genotype prevalence by endemicity.

**Figure 4:**
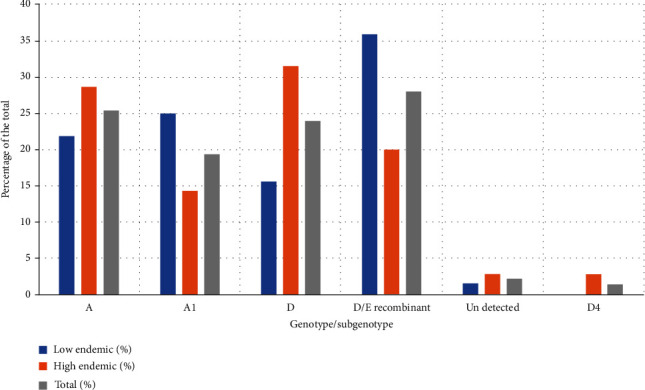
Relative proportion of the different genotypes and subgenotypes in the low and high endemic regions of Uganda.

In general, 60 (46%) of the samples sequenced belonged to genotype A; 37 (28%) were for the recombinant genotype D/E while 34 (26%) were for genotype D. According to the endemicity and genotype distribution, genotype A was slightly more prevalent in the low endemic region (47.6%) compared to the high endemic (44.1%). In contrast, genotype D was the most common in the high endemic region (38.0%) compared to the low endemic (15.9%) while for the recombinant genotype D/E, it was proportionately higher in the low endemic region (36.5%) than in the high endemic region (20.6%) (Figure 3). Regarding the distribution of genotype and subgenotype in relation to the endemicity genotypes, A was predominantly circulating in the high endemic region with a prevalence of 20 (58.8%) compared to the low endemic region with a prevalence of 14 (41.2%). Similarly, genotype D was proportionately higher among the patients from the high endemic region at a prevalence rate of 22 (68.8%) compared to those from the low endemic region at a prevalence rate of 10 (15.6%). In contrast, subgenotype A1 was dominating in the low endemic region at a prevalence rate of 16 (61.5%) compared to the high endemic region at a prevalence of 10 (38.5%) and only two cases of subgenotype D4 from the high endemic region were detected. Only four samples (3.0%) failed to give sequences (Table 2, Figure 4).

### 3.4. Variation in the Genotype Distribution with Endemicity and Associated Sociodemographic Factors

We performed the bivariable analysis using the Pearson chi-square to select candidate variables for the final model of multinomial logistic regression analysis. All variables with a *p* < 0.05 in the bivariate analysis were selected for the final model of analysis. As presented at the bivariate level (Supplementary material S3, Table B), genotype distribution differed significantly by endemicity and age (*p* < 0.05) but not with the other sociodemographic factors including sex, marital status, alcohol used, birth place, previous infection with an STD, and household contact with an infected person (*p* > 0.05). In the multinomial logistic regression analysis model, our study has established that the hepatitis B virus chronically infected persons from the high endemic region were significantly more likely to be infected with genotype D compared to the inhabitants of the low endemic region (AOR = 4.189, 95%CI = [1.44 to 12.18], *p* = 0.009). Thus, genotype D was 4.2 times more likely to be circulating in the high endemic region compared to the low endemic region. On the other hand, though the distribution of genotype A between the low and high endemic regions did not differ significantly, it was almost 2 times more likely to be found in the low endemic region (AOR = 1.697, 95%CI = [0.72 to 4.0]) (Table 3).

As presented in Table 4, prevalence of genotypes A did not differ by age group (*p* > 0.05). In contrast, the prevalence of genotype D has a significantly higher proportion among the elderly (AOR = 9.98, 95% = [1.696 to 58.8], *p* = 0.011) compared to other age groups. Thus, our results have shown that persons aged 50 years and above were 10 times more likely to be infected with genotype D (Table 5).

Bivariable analysis was also conducted using Pearson's chi-square on the variations in markers of liver damage and viral load with genotypes to establish potential variables for multinomial logistic regression analysis as previously described (Supplementary material S4, Table C). Again, variables with *p* < 0.05 in bivariate analysis were maintained for analysis in the final model.

Basing on the aforesaid criterion, GGT (*X*^2^ = 8.425, *p* = 0.015), direct bilirubin (*X*^2^ = 6.9, *p* = 0.032), and viral load (*X*^2^ = 31.7, *p* < 0.000) merited analysis at a higher level of multinomial logistic regression model. In this model, persons infected with genotype A presented with significantly elevated GGT (AOR = 6.06, 95%CI = [2.19 to 16.81], *p* = 0.001). Thus, infection with genotype A was 6 times more associated with elevated GGT. In contrast, infection with genotype A was marked by reduced odds of elevated direct bilirubin (AOR = 0.167, 95%CI = [0.07 to 0.499], *p* = 0.001) and reduced odds of the viral load exceeding the 20.000 copies/mL threshold for treatment eligibility (AOR = 0.146, 95%CI = [0.047 to 0.45], *p* = 0.001). Therefore, the risk of having elevated bilirubin when infected with genotype A relative to the recombinant E/D was only 16.7%. Similarly, the risk of having a viral load of greater than 20,000 copies/mL when infected with genotype A was only 14.6% (Table 6). Finally, infection with the recombinant genotype D/E was significantly associated with elevated viral load (AOR = 3.77, 95%CI = [1.32 to 10.756], *p* = 0.013). Hence, persons infected with the recombinant genotype D/E were almost 4 times more likely to have elevated viral load (Table 7).

### 3.5. Variations in the Levels of the Liver Function Test Parameters and Viral Load with Genotypes A and D for All the Study Participants

**Figure 5:**
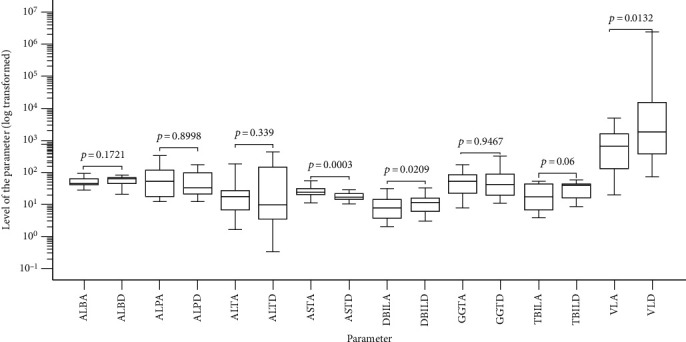
Box and Whisker plot showing the variation s in the levels of the clinical parameter investigated with the genotypes A and D: ALBA: albumin for genotype A; ALBD: albumin for genotype D; ALPA: alkaline phosphatase for genotype A; ALTA: alanine aminotransferase for genotype A; ALTD: alanine aminotransferase for genotype D; ASTA: aspartate aminotransferase for genotype A; ALTD: aspartate aminotransferase for genotype D; DBIL: direct bilirubin for genotype A; DBILD: direct bilirubin for genotype D; GGTA: gamma glutamyl transferase for genotype A; GGTD gamma glutamyl transferase for genotype D; TBILA: total bilirubin for genotype A; TBILD: total bilirubin for genotype D; VLA: viral load for genotype A; VLD: viral load for genotype D.

We compared relative variations in viral load and the levels of liver function evaluation parameters among hepatitis B virus chronically infected persons with genotypes A and D using the nonparametric Mann–Whitney *U* test due to overall lack of normality in the data (Supplementary material S5, Table D). Genotype A was associated with significantly elevated AST (*p* = 0.0003). On the other hand, genotype D was significantly associated with elevated direct bilirubin (*p* = 0.0209) and elevated viral load (*p* = 0.0132) (Figure 5).

## 4. Discussion

Hepatitis B virus genotypes, subgenotypes, and recombinant genotypes are key determinants of viral evolution as well as tracing the HBV transmission patterns in communities. Consequently, routine surveillance of the circulating HBV genotypes, their recombinants, or the subgenotypes should be of prime attention if the UN/WHO target of eliminating HBV by 2030 is to be realized. This research was aimed at establishing the circulating hepatitis B virus genotypes in a low and a high endemic region of Uganda in order to give a plausible explanation for the regional differences in disease burden. The nucleotide sequence analysis of the hepatitis B virus DNA polymerase gene in all patient samples from the low and high endemic regions detected genotypes A and D consistent with earlier studies in other African countries including Tunisia [27], Gambia, Nigeria, Congo, Rwanda, Cameroon [34], Egypt [35], Central African Republic [36], South Africa [20], Morocco [37], Kenya [38], and Uganda [9]. Besides, a high proportion of the D/E recombinant genotype mildly reported in Uganda by Zirabamuzaale and Ocama [9] has been prominently reported in our study. These recombinant genotypes have been earlier reported in Guinea [39] and were closely related to genotype D8 reported in Niger [40]. This discrepancy in the prevalence of D/E recombinant genotype reported in our study compared to the previous studies can be explained by the intercontinental and continental travels [41]. In addition, our study has revealed a high proportion of subgenotype A1 not earlier reported in Uganda but previously reported in Central African Republic [36] and the neighboring Kenya [38, 42]. Besides, genotype A subgenotype A1 has been reported in previous studies to be dominant in eastern, southern, and central Africa in conformity with the results of our study [43]. Consistent with previous studies in Uganda and elsewhere in Africa, genotypes B, C, F, G, H, and J are not frequently reported [27] and were as well not detected in our study. One novel subgenotype D4 earlier reported in Central African Republic [36], Kenya among blood donors [44], Rwanda in the general population [45], and from South Africa [46] was sporadically identified in the current study. Conspicuously, genotype E was missing among the circulating HBV strains both in the low and high endemic regions despite its predominance in may African countries including the West Africa coastal countries [47] and the neighboring Democratic Republic of Congo (DRC) [48]. Thus, Uganda appears not to be part of the massive African genotype E corridor.

Genotype A is associated with sexual transmission whereas genotype D with blood transfusion [24, 49]. The predominance of these genotypes suggests an unmet need pertaining safe sex practices and safe blood transfusion despite the recommendation by the WHO to screen the donated blood for HBV prior to transfusion into the recipient [50]. Thus, the recommendations by the WHO pertaining rigorous screening of blood before any transfusion should be implemented by the Ugandan Ministry of Health. In addition, our data suggests that sex education which promotes safe sex practices should be introduced in schools and in public health awareness campaigns to reduce cases of sexually transmitted hepatitis B virus infections. Finally, cultural and religious institutions should be part of the campaigns against promiscuity.

Our study has revealed genotypes A and D to be circulating in Uganda but disproportionately distributed in the low and high endemic regions with both of them predominating in the high endemic region. On the other hand, subgenotype A1 and the D/E recombinant genotypes were dominating in the low endemic region. These genotypes present with unique disease severity, prognosis, and response to antiviral therapy. Genotype A has been reported to be frequently associated with progression to chronic infection and HCC in an African population compared to other genotypes and an increased likelihood of resistance to nucleotide/nucleoside analog antivirals but better response to interferons [16]. Similarly, subgenotype A1 has been associated with high HBV replication levels and rapid emergency of antiviral drug resistance in Malawi [51]. So, there is an increased risk of developing chronic disease that is resistant to nucleotide/nucleoside analog antiviral drugs as well as an increased risk of hepatocellular carcinogenicity as earlier reported [12]. Hence, in managing the chronic HBV patients in our cohort, pegylated interferon should be the drug of choice due to better response among patients infected with genotype A and subgenotype A1. Besides, earlier studies have associated genotype A and the subgenotype A1 to more severe disease among the Bantu-speaking South African people compared to non-Bantu speakers [16]. This is contrary to the findings of our study probably because of migrations and intermarriages among Ugandans. Nonetheless, more studies are needed using large sample sizes for a more comprehensive conclusion on the relationship between ethnicity and genotype preference. Genotype D is associated with fulminant hepatitis, severe liver diseases compared to other genotypes [52], and precore mutation increasing the risk of liver cirrhosis and HCC [53]. Thus, our results suggest that precore mutations are likely to be more common among the chronic HBV-infected patients in the high endemic region increasing the risk of developing liver cirrhosis and HCC. However, studies on HBV whole genome sequence analysis to confirm the presumed mutations in the precore exon are needed.

Pertaining to the variations in viral load with infecting genotype or recombinant genotype, a high viral load was reported among HBV D/E recombinant genotype-infected patients than those infected with either genotype A or D. This is in conformity with the earlier findings [21, 43]. Moreover, the D/E recombinant genotype has been implicated in high infectivity of the HBV [43]. Furthermore, when we compared the viral load among the patients infected with genotypes A and D, those infected with the later genotype presented with significantly higher viral load consistent with the findings from other studies [17–20].

Regarding age and relative genotype distribution, studies elsewhere have associated age and HBV infection with a significant increase in HBV seropositivity among persons of higher age [54]. This has been attributed to mutations in the B-cell and T-cell epitopes in the S-gene of the HBV genome that may lead to breakthrough infections even among the vaccinated [55] as well as differences in the infecting genotype [56]. We did not observe a significant association between genotype A and age in conformity with the findings from other studies [57, 58]. However, Muriel et al. [56] observed a significantly increased risk of infection with genotype A among the persons aged between 29 and 41 years. The differences in our findings and those from the aforementioned study could be attributed to differences in circulating genotypes, behavioral factors, and host genetic factors as well as differences in the implementation of the control measures against HBV. In our study, the prevalence of genotype D was significantly higher among the participants aged 50 years and above compared to the recombinant genotype D/E. The high prevalence of genotype D among the elderly would suggest fulminant hepatitis, precore mutations in this age group, and severe liver diseases in this age group [52, 53].

Finally, whereas most clinical chemistry parameters did not vary significantly with genotypes in conformity with the report by Muriel et al. [56], *γ*-GT was significantly elevated among chronically infected HBV patients with genotype A. The elevations in *γ*-GT observed among the subjects infected with D/E recombinant genotypes could be predictive of liver-related damage as it is associated with high viral load giving presumptive diagnosis of HCC [59]. However, sclerosing cholangitis, cholecystitis, and alcoholism have also been implicated in causing elevations in *γ*-GT giving false-positive rate when *γ*-GT is used alone [60, 61]. Moreover, the levels of *γ*-GT did not differ significantly between genotypes A and D (*p* > 0.05). Thus, implicating *γ*-GT elevation to a particular genotype may be unrealistic. However, large-scale studies are recommended to justify our observations. On the other hand, direct bilirubin was significantly normal among those infected with genotype A but did not differ significantly among those infected with genotype D using D/E as the reference variable. Thus, the liver is still capable of conjugating bilirubin suggesting a lower aggressive nature of genotype A on the hepatocytes compared to genotypes D [19], B, and C [62]. The AST was significantly elevated among patients infected with genotype A compared to those infected with genotype D. The AST is both cytosolic (20%) and mitochondrial (80%) [63]. Thus, it is plausible that the AST rate goes up in chronic cases because the mitochondria are damaged at a later stage as opposed to an earlier cytoplasmic damage that manifests in the acute phase of the infection. Moreover, genotype A has been closely linked to chronic hepatitis B virus infection compared to genotype D [16]. Nonetheless, AST is unspecific to viral hepatitis, and its elevation has been concomitantly associated with alcohol hepatitis and drug-induced hepatitis [64]. Besides, serum AST may originate from the heart, skeletal muscles, and the brain [65]. Hence, implicating the elevation of ALT on genotype A would be idealistic.

In conclusion, available information on HBV genotypes and their consequence on clinical profile during infection is at present limited to specific genotypes particularly A, B, C, and D. Nonetheless, subgenotypes and recombinant genotypes also play a key role in disease profile. Thus, in addition to genotype determination, subgenotype and genotype recombinants should be determined. This would help in identifying the patients who are at a risk of disease progression and establishing the candidate treatment options. Despite using samples collected from outpatients, our study has highlighted novel recombinant genotype D/E and subgenotype A1 in proportionately large numbers not earlier reported in Uganda. We therefore recommended routine genotyping to establish the circulating genotypes, recombinant genotypes, and subgenotypes for the effective management of HBV in our country.

### 4.1. Limitations of the Study

The study was limited by the sample size from which sequences for HBV genotyping could be obtained. Nonetheless, the study has given constructive clues on the circulating genotypes in the low and high endemic regions which can provide a basis for future research on the subject particularly in the area of patient care. Furthermore, the genotyping platform could not identify other subgenotypes of genotypes A and D. The short fragment (400 bp) sequence could have contributed to the failure to identify a wide array of subgenotypes. Therefore, whole genome sequencing is certainly required for accurate classification of the HBV genotypes and subgenotypes. Moreover, no downstream validation programs were performed to assess the accuracy of the estimated frequency of genotypes, subgenotypes, and recombinant genotypes in individual samples based on genotyping by sequencing (GBS) data. Finally, we did not investigate the influence of HIV coinfection and ARV treatment on the circulating genotypes. This could have limited the information richness obtainable from our findings. Therefore, future research should focus on comprehensive evaluation of the circulating HBV genotypes in areas of low, moderate, and high endemicity using a large sample size in order to account for the differential disease burden by region and guide the implementation of region-specific public health interventions.

## Figures and Tables

**Table 1:** Sociodemographic characteristics of study participants in a low and high endemic region with detectable HBV DNA.

Variable	Category	*N*	Marginal percentage
Endemicity	High	70	51.9%
	Low	65	48.1%
Genotype	A	34	25.2%
	A1	26	19.3%
	D	32	23.7%
	D4	2	1.5%
	D/E	37	27.4%
	ND	4	3.0%
Sex	Female	85	63.0%
	Male	50	37.0%
Age (years)	≥50	19	14.1%
	31-40	33	24.4%
	41-50	21	15.6%
	18-30	62	45.9%
Marital status	Divorced	15	11.1%
	Married	86	63.7%
	Single	31	23.0%
	Widowed	3	2.2%
Education level	Post-secondary	19	14.07%
	Primary	50	37.0%
	Secondary	43	31.9%
	Unknown	23	17.0%
Total		135	100.0%

Abbreviation: ND: not detected.

**Table 2:** Distribution of HBV genotypes and subgenotypes in the low and high endemic regions.

			Genotype/subgenotype/recombinant genotype						Total
			A	A1	D	D4	D/E	ND	
Endemicity	High	Count	20	10	22	2	14	2	70
		%	58.8	38.50	68.8	100.0	37.8	50.0	51.9
	Low	Count	14	16	10	0	23	2	65
		%	41.2	61.50	31.3	0.0	62.2	50.0	48.1
Total		Count	34	26	32	2	37	4	135
		%	100.0	100.0	100.0	100.0	100.0	100.0	100.0

Abbreviation: ND: not detected.

**Table 3:** Multilevel analysis of genotype distribution in the low and high endemic regions using multinomial regression analysis.

Variable	Categories	Endemicity		COR [95% CI]	AOR [95% CI]	*p* value
		High	Low			
Genotype	D/E^∗∗^	14 (20.6)	23 (36.5)	1	1	
	A	30 (44.1)	30 (47.6)	1.64 [0.71 to 3.79]	1.697 [0.72 to 4.0]	0.227
	D	24 (35.3)	10 (15.9)	3.94 [1.46 to 10.64]	4.189 [1.44 to 12.18]	0.009^∗^

^∗∗^Reference genotype according to the model used for analysis; COR: crude odds ratio; AOR: adjusted odds ratio; ^∗^*p* < 0.05 significant at 95% confidence interval.

**Table 4:** Variation between age and genotype A distribution using multinomial regression analysis.

Variable	Categories	Genotype		COR [95% CI]	AOR [95% CI]	*p* value
		A	D/E^∗∗^			
Age (years)	18-30	33 (54.1)	18 (29.5)	1	1	
	31-40	12 (37.5)	8 (25.0)	0.818 [0.283 to 2.37]	0.75 [0.26 to 2.2]	0.602
	41-49	8 (40.0)	9 (45.0)	0.485 [0.159 to 1.474]	0.50 [0.164 to 1.54]	0.230
	≥50	7 (38.9)	2 (11.1)	1.91 [0.358 to 10.173]	2.06 [0.38 to 11.05]	0.401

^∗∗^Reference genotype according to the model used for analysis; COR: crude odds ratio; AOR: adjusted odds ratio.

**Table 5:** Variation between age and genotype D distribution using multinomial regression analysis.

Variable	Categories	Genotype		COR [95% CI]	AOR [95% CI]	*p* value
		D	D/E^∗∗^			
Age (years)	18-30	10 (16.4)	18 (29.5)	1	1	
	31-40	12 (37.5)	8 (25.0)	2.7 [0.828 to 8.807]	2.178 [0.64 to 7.4]	0.213
	41-49	3 (15.0)	9 (45.0)	0.60 [0.131 to 2.738]	0.664 [0.14 to 3.18]	0.608
	≥50	9 (50.0)	2 (11.1)	8.10 [1.46 to 45.06]	9.98 [1.696 to 58.8]	0.011^∗^

^∗∗^Reference genotype according to the model used for analysis, COR: crude odds ratio, AOR: adjusted odds ratio; ^∗^*p* < 0.05 significant at 95% confidence interval.

**Table 6:** Analysis of the variation in the levels of GGT, DBIL, and VL with genotype A using multinomial logistic regression analysis.

Variable	Categories	Genotype		COR [95% CI]	AOR [95% CI]	*p* value
		A, *n* (%)	D/E^∗∗^, *n* (%)			
GGT (U/L)	Normal	24 (35.8)	26 (38.8)	1	1	
	Elevated	36 (58.1)	11 (17.7)	3.545 [1.48 to 8.496]	6.06 [2.19 to 16.81]	0.001^∗^
DBIL (*μ*mol/L)	Normal	29 (61.7)	10 (21.3)	1	1	
	Elevated	31 (37.8)	27 (32.9)	0.396 [0.163 to 0.959]	0.167 [0.07 to 0.499]	0.001^∗^
VL (IU/mL)	<20,000	52 (62.7)	22 (26.5)	1	1	
	≥20,000	8 (17.4)	15 (32.6)	0.226 [0.084 to 0.609]	0.146 [0.047 to 0.45]	0.001^∗^

^∗∗^Reference genotype according to the model used for analysis; COR: crude odds ratio; AOR: adjusted odds ratio; GGT: gamma glutamyl transferase; DBIL: direct bilirubin; VL: viral load. ^∗^*p* < 0.05 significant at 95% confidence interval.

**Table 7:** Analysis of the variation in the levels of GGT, DBIL, and VL with genotype D using multinomial logistic regression analysis.

Variable	Categories	Genotype		COR [95% CI]	AOR [95% CI]	*p* value
		D, *n* (%)	D/E^∗∗^, *n* (%)			
GGT (U/L)	Normal	17 (25.4)	26 (38.8)	1	1	
	Elevated	15 (24.2)	11 (17.7)	2.09 [0.775 to 5.61]	1.82 [0.625 to 5.296]	0.272
DBIL (*μ*mol/L)	Normal	8 (17.0)	10 (21.3)	1	1	
	Elevated	24 (29.3)	27 (32.9)	1.11 [0.377 to 3.272]	1.26 [0.384 to 4.137]	0.702
VL (IU/mL)	<20,000	23 (50.0)	22 (26.5)	1	1	
	≥20,000	9 (10.8)	15 (32.6)	3.748 [1.36 to 10.31]	3.77 [1.32 to 10.756]	0.013∗

^∗∗^Reference genotype according to the model used for analysis; COR: crude odds ratio; AOR: adjusted odds ratio; GGT: gamma glutamyl transferase; DBIL: direct bilirubin; VL: viral load. ^∗^*p* < 0.05 significant at 95% confidence interval.

## Data Availability

All data generated or analyzed during this study are included in this published article. The data on the HBV genotype NCBI accession numbers is available on request from the principal investigator (PI) on the following email: hsendagire@yahoo.com. This data will be made public when all the downstream molecular analyses of the HBV genome including the precore and S-gene mutations are completed.

## Abbreviations

ALP: Alkaline phosphatase
ALT: Alanine aminotransferase
CPHIL: Central Public Health Laboratories
CPHL: Public Health Reference Laboratories
GGT: *γ*-Glutamyl transferase
HBsAg: Hepatitis B Surface Antigen
HBV: Hepatitis B virus
HCC: Hepatocellular carcinoma
MTCT: Mother-to-child transmission
UPHIA: Uganda Population-Based HIV-Impact Assessment
GBS: Genotyping by sequencing
NCBI: National Center for Biotechnology Information
ARV: Antiretroviral

## Supplementary Materials

Supplementary Materials Supplementary material S1 Table A: sociodemographic characteristics of all the participants screened for HBsAg seropositivity from both the high and low endemic regions. Supplementary material S2, Figures A and B: A: 2% agarose gel electrophoresis of the nested PCR products. Lane M: DNA 100 bp marker (Amersham Biosciences, UK); lanes 1-3, 6, 12, and 14-18 are positive samples for the HBV pol gene amplicons for the low endemic region. Lanes 4,5, 7-11, and 13 are negative samples for the HBV pol gene. B: 2% agarose gel electrophoresis of the nested PCR products. Lane M, DNA 100 bp marker (Amersham Biosciences, UK); lanes 5, 6, 8-11, and 15-17 are HBV pol gene amplicons for the high endemic region. Lanes 1-4, 7, 12-14, and 18 are negative samples for the HBV pol gene. Supplementary material S3, Table B: analysis of genotype distribution in the high and low endemic regions and the associated risk factors using Pearson's chi-square analysis. Supplementary material S4, Table C: analysis of genotype distribution in the high and low endemic regions and the associated risk factors using Pearson's chi-square analysis. Supplementary material S5, Table D: summary statistics for the clinical parameters and the test for normality using D'Agostino Pearson test.
