# Supplementary material for: Disproportionate Distribution of HBV Genotypes A and D and the Recombinant Genotype D/E in the High and Low HBV Endemic Regions of Uganda: A Wake-Up Call for Regional Specific HBV Management
Source: Int J Hepatol. 2022 Jan 11;2022:3688547. doi: 10.1155/2022/3688547 (PMC8767397; doi:10.1155/2022/3688547)
Supplement: Supplementary Materials — Supplementary material S1 Table A: sociodemographic characteristics of all the participants screened for HBsAg seropositivity from both the high and low endemic regions. Supplementary material S2, Figures A and B: A: 2% agarose gel electrophoresis of the nested PCR products. Lane M: DNA 100 bp marker (Amersham Biosciences, UK); lanes 1-3, 6, 12, and 14-18 are positive samples for the HBV pol gene amplicons for the low endemic region. Lanes 4,5, 7-11, and 13 are negative samples for the HBV pol gene. B: 2% agarose gel electrophoresis of the nested PCR products. Lane M, DNA 100 bp marker (Amersham Biosciences, UK); lanes 5, 6, 8-11, and 15-17 are HBV pol gene amplicons for the high endemic region. Lanes 1-4, 7, 12-14, and 18 are negative samples for the HBV pol gene. Supplementary material S3, Table B: analysis of genotype distribution in the high and low endemic regions and the associated risk factors using Pearson's chi-square analysis. Supplementary material S4, Table C: analysis of genotype distribution in the high and low endemic regions and the associated risk factors using Pearson's chi-square analysis. Supplementary material S5, Table D: summary statistics for the clinical parameters and the test for normality using D'Agostino Pearson test. [file 3688547.f1.docx]

Supplementary material S1 Table A**:** Socio-demographic characteristics of all the participants screened for HBsAg sero-positivity from both the high and low endemic regions.

| Variable Category | | Frequency | Marginal Percentage |
| --- | --- | --- | --- |
| Endemicity | High | 99 | 49.5% |
|  | Low | 101 | 50.5% |
| Sex | Female | 129 | 64.5% |
|  | Male | 71 | 35.5% |
| Age | ≥50 | 30 | 15.0% |
|  | 40-49 | 26 | 13.0% |
|  | 31-39 | 42 | 21.0% |
|  | 18-30 | 102 | 51.0% |
| Marital status | Divorced | 19 | 9.5% |
|  | Married | 124 | 62.0% |
|  | Single | 51 | 25.5% |
|  | Widowed | 6 | 3.0% |
| Education level | Post-Secondary | 24 | 12.0% |
|  | Primary | 81 | 40.5% |
|  | Secondary | 58 | 29.0% |
|  | Unknown | 37 | 18.5% |
| Total | | 200 | 100.0% |

**Supplementary material S2, Figures A and B: -** A: 2% agarose gel electrophoresis of the nested PCR products. Lanes M, DNA 100bp marker (Amersham Biosciences, UK), lanes 1-3,6,12,14-18 are positive samples for the HBV pol gene amplicons for the low endemic region. Lanes 4,5, 7-11 and 13 are negative samples for the HBV pol gene**.** B: 2% agarose gel electrophoresis of the nested PCR products. Lanes M, DNA 100bp marker (Amersham Biosciences, UK), lanes 5,6,8-11, 15-17 are HBV pol gene amplicons for the high endemic region. Lanes 1-4, 7, 12-14, and 18 are negative samples for the HBV pol gene.


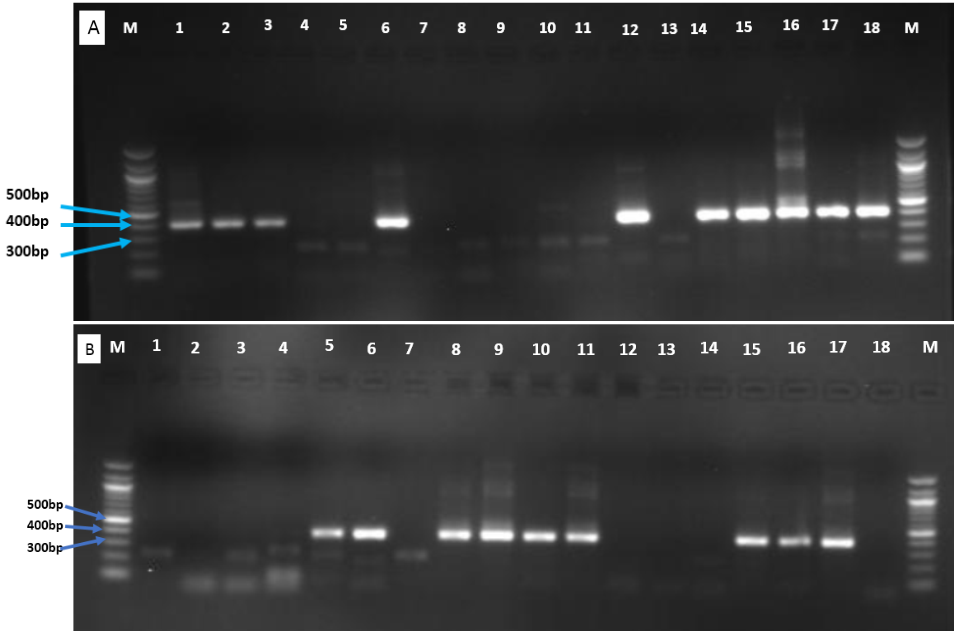


Supplementary material S3, Table B*:* Analysis of genotype distribution in the high and low endemic regions and the associated risk factors using Pearson’s Chi-square analysis

| **Variable** | **Categories** | **Genotype** | |  | **Pearson's Chi square** | **Pearson's Chi p-value** |
| --- | --- | --- | --- | --- | --- | --- |
|  |  | **A** | **D** | **D/E** |  |  |
| Endemicity | High | 30(44.1) | 24(35.3) | 14(20.6) |  |  |
|  | Low | 30(47.6) | 10(15.9) | 23(36.5) | 7.774 | 0.021* |
|  |  |  |  |  |  |  |
| Age (Years) | 18-30 | 33(54.1) | 10(16.4) | 18(29.5) |  |  |
|  | 31-40 | 12(37.5) | 12(37.5) | 8(25.0) |  |  |
|  | 41-49 | 8(40.0) | 3(15.0) | 9(45.0) |  |  |
|  | ≥50 | 7(38.9) | 9(50.0) | 2(11.1) | 14.472 | 0.025* |
|  |  |  |  |  |  |  |
| Sex | Female | 39(47.0) | 22(26.5) | 22(26.5) |  |  |
|  | Male | 21(43.8) | 12(25.0) | 15(31.3) | 0.339 | 0.844 |
|  |  |  |  |  |  |  |
| Marital status | Married | 39(47.0) | 22(26.5) | 22(26.5) |  |  |
|  | Not married | 21(43.8) | 12(25.0) | 31.3(48) | 0.34 | 0.840 |
|  |  |  |  |  |  |  |
| Alcohol use | Yes | 12(45.8) | 8(26.7) | 10(33.3) |  |  |
|  | No | 48(47.5) | 26(25.7) | 27(26.7) | 0.65 | 0.722 |
|  |  |  |  |  |  |  |
| Born from health facility | Yes | 34(42.0) | 20(24.7) | 27(33.3) |  |  |
|  | No | 26(52.0) | 14(28.0) | 10(20.0) | 2.755 | 0.252 |
|  |  |  |  |  |  |  |
| STD treatment | Yes | 19(50.0) | 8(21.1) | 11(28.9) |  |  |
|  | No | 41(44.1) | 26(28.0) | 26(28.0) | 0.711 | 0.701 |
|  |  |  |  |  |  |  |
| Contact with HBV infected person | Yes | 13(41.9) | 8(25.8) | 10(32.3) |  |  |
|  | No | 47(47.0) | 26(26.0) | 27(27.0) | 0.365 | 0.833 |

*p<0.05 significant at 95% confidence interval, STD: sexually transmitted disease

Supplementary material S4, Table C: Analysis of genotype distribution in the high and low endemic regions and the associated risk factors using Pearson’s Chi-square analysis

| **Variable** | **Categories** | **Genotype** | |  | **Pearson's Chi square** | **Pearson's Chi p-value** |
| --- | --- | --- | --- | --- | --- | --- |
|  |  | **A, n (%)** | **D, n (%)** | **D/E, n (%)** |  |  |
| AST (U/L) | Elevated | 31(43.7) | 19(26.8) | 21(29.6) |  |  |
|  | Normal | 29(50.0) | 13(22.4) | 16(27.6) | 0.563 | 0.755 |
|  |  |  |  |  |  |  |
| ALP (U/L) | Elevated | 14(53.8) | 7(26.9) | 5(19.2) |  |  |
|  | Low | 24(49.0) | 13(26.5) | 12(24.5) |  |  |
|  | Normal | 22(40.7) | 12(22.2) | 20(30.7) | 3.427 | 0.489 |
|  |  |  |  |  |  |  |
| GGT (U/L) | Elevated | 36(58.1) | 15(24.2) | 11(17.7) |  |  |
|  | Normal | 24(35.8) | 17(25.4) | 26(38.8) | 8.425 | 0.015* |
|  |  |  |  |  |  |  |
| ALB (g/L) | Elevated | 24(38.7) | 21(33.9) | 17(27.4) |  |  |
|  | Low | 4(30.8) | 4(30.8) | 5(38.5) |  |  |
|  | Normal | 32(59.3) | 7(13.0) | 15(27.8) | 9.167 | 0.057 |
|  |  |  |  |  |  |  |
| TBIL (µmol/L) | Elevated | 30(46.9) | 20(31.3) | 14(21.9) |  |  |
|  | Normal | 30(46.2) | 12(18.5) | 23(35.4) | 4.182 | 0.124 |
|  |  |  |  |  |  |  |
| DBIL (µmol/L) | Elevated | 31(37.8) | 24(29.3) | 27(32.9) |  |  |
|  | Normal | 29(61.7) | 8(17.0) | 10(21.3) | 6.9 | 0.032* |
|  |  |  |  |  |  |  |
| ALT (U/L) | Elevated | 9(32.1) | 7(25.0) | 12(42.9) |  |  |
|  | Normal | 51(50.5) | 25(24.8) | 25(24.8) | 4.093 | 0.129 |
|  |  |  |  |  |  |  |
| Viral load (IU/mL) | Above 20,000 | 8(17.4) | 23(50.0) | 15(32.6) |  |  |
|  | Below 20,000 | 52(62.7) | 9(10.8) | 22(26.5) | 31.71 | 0.000* |

AST: aspartate aminotransferase, ALP: alkaline phosphatase, GGT: gamma glutamyl transferase, ALB: albumin, TBIL: total bilirubin, DBIL: direct bilirubin, ALT: alanine aminotransferase

Supplementary material S5, Table D: Summary statistics for the clinical parameters and the test for normality using D’Agostino Pearson test.

| **Variable** | **N** | **Min** | **Max** | **Mean** | **SD** | **Normal Distr.** |
| --- | --- | --- | --- | --- | --- | --- |
| ALB (U/L) | 135 | 12.3 | 99.8 | 53.984 | 18.6007 | 0.0941 |
| ALP (U/L) | 135 | 11.1 | 335.6 | 70.984 | 60.5671 | <0.0001 |
| ALT (U/L) | 135 | 0.34 | 442.8 | 54.6 | 84.7393 | <0.0001 |
| AST (U/L) | 135 | 10.6 | 149.5 | 24.678 | 20.0388 | <0.0001 |
| DBIL (µmol/L) | 135 | 2.04 | 33.53 | 11.586 | 8.0343 | <0.0001 |
| GGT (U/L) | 135 | 8.1 | 328.1 | 62.666 | 58.7829 | <0.0001 |
| TBIL (µmol/L) | 135 | 3.9 | 58.1 | 26.338 | 17.4572 | <0.0001 |
| VL (IU/mL) | 135 | 20 | 170000000 | 7764602.99 | 35126130.92 | <0.0001 |
